# Detection of T-Cadherin Expression in Mouse Embryos

**Published:** 2015

**Authors:** K. A. Rubina, V. A. Smutova, M. L. Semenova, A. A. Poliakov, S. Gerety, D. Wilkinson, E. I. Surkova, E. V. Semina, V. Yu. Sysoeva, V. A. Tkachuk

**Affiliations:** Department of Biochemistry and Molecular Medicine, Faculty of Fundamental Medicine, Lomonosov Moscow State University, Lomonosovskiy Prosp., 31/5, 119192, Moscow, Russia; Department of Embryology, Biology Faculty, Lomonosov Moscow State University, Leninskie Gory, 1/12, 119234, Moscow, Russia; Division of Developmental Neurobiology, MRC National Institute for Medical Research, The Ridgeway, Mill Hill, London NW7 1AA, UK; Wellcome Trust Sanger Institute, Wellcome Trust Genome Campus, Hinxton, Cambridge CB10 1SA, UK

**Keywords:** T-cadherin, embryogenesis, angiogenesis, in situ hybridization

## Abstract

The aim of the present study was to evaluate T-cadherin expression at the early developmental stages of the mouse embryo. Using *in situ *hybridization and immunofluorescent staining of whole embryos in combination with confocal microscopy, we found that T-cadherin expression is detected in the developing brain, starting with the E8.75 stage, and in the heart, starting with the E11.5 stage. These data suggest a possible involvement of T-cadherin in the formation of blood vessels during embryogenesis.

## INTRODUCTION

T-cadherin was first discovered in a chick embryo brain over 20 years ago [1]. In early studies, Ranscht demonstrated [1] that expression of T-cadherin in developing somites correlates with migration of neural crest cells from the neural tube. Neural crest cells are the transient multipotent population of cells that further give rise to a variety of tissues, including craniofacial bones and cartilages, smooth muscle cells, melanocytes, peripheral neurons, glia, etc. More recent studies by the same laboratory have revealed that migratory neural crest cells and motor neuron axons growing to their targets choose their pathway through the rostral part of the somites, avoiding, along their pathway, the caudal part of the somites where cells express T-cadherin.* In vitro *experiments, using soluble T-cadherin or T-cadherin as a substrate, demonstrated that T-cadherin inhibits the development of neurites and growth of motor neuron axons. This suggested that T-cadherin functions as a guidance molecule for growing axons and migrating neural crest cells [2, 3]. Like other guidance molecules, ephrins and their receptors [4], T-cadherin in the developing nervous system acts as a “repulsive molecule” and negatively regulates axon growth and cell migration.

It should be noted that the T-cadherin expression level in an adult brain is higher than that in an embryonic brain [5]. Our laboratory found that T-cadherin in an adult organism is expressed not only in the nervous system, but also in the cardiovascular system [6, 7]. Immunohistochemical staining of aorta sections revealed the presence of T-cadherin in all layers of the vascular wall (intima, media, and adventitia), endothelium, smooth muscle cells, and pericytes. A high level of T-cadherin in the adventitia was detected in the *vasa vasorum *walls [6]. We also observed elevated T-cadherin expression in blood vessels in various pathologies: upon development of atherosclerotic lesions and post-angioplasty restenosis, which are conditions associated with pathological angiogenesis in humans [6, 7]. Furthermore, overexpression of T-cadherin in the arterial wall after balloon angioplasty in rats was found to correlate with late stages of the neointima formation and to coincide with the phase of active migration and proliferation of vascular cells. Expression of T-cadherin in the *vasa vasorum *of the adventitia of damaged blood vessels suggests its involvement in the regulation of angiogenesis or repair of vascular wall damage [7].

The formation of the nervous and cardiovascular systems during embryogenesis is known to occur in parallel, whereby nerves and blood vessels are often located in close proximity to each other. Nerve and vascular cells secrete neurotrophic and angiogenic factors, respectively, which promote their survival and determine the direction of growth and migration [8]. The regulatory mechanisms of directed axonal growth and nerve cell migration are well studied [4, 9, 10], while there is much less data on the factors and mechanisms regulating directed growth of blood vessels. Guidance molecules that are involved in the regulation of the formation of the nervous and vascular systems include proteins such as semaphorins and their receptors (plexins and neuropilins), netrins and their receptors (DCC/neogenin and Unc5), slit ligands and their receptors (Robo), and some other proteins [10]. Data on the expression of T-cadherin in the cardiovascular system during embryogenesis have yet to be reported. It is unknown whether T-cadherin is expressed in the developing heart and blood vessels during embryogenesis, or whether its role is limited to the regulation of the trajectory of axon growth and migration of neural crest cells.

In this regard, we analyzed the expression of T-cadherin at different stages of a mouse’s development using* in situ *hybridization and immunofluorescent staining of whole embryos in combination with confocal microscopy. Expression of the T-cadherin mRNA was detected starting with the E8.75 stage in the developing brain and starting with the E11.5 stage in the heart, which coincides with the processes of active growth and formation of blood vessels due to vasculogenesis and angiogenesis in the cardiovascular system and brain.

## EXPERIMENTAL

**Production of dated pregnancy in mice**

Mouse embryos derived from F1 CBA/C57Bl6 hybrids were used in this study. Mice were maintained under standard, 14-hour light conditions. In the evening hours, males were introduced to females, and, the next morning, mated mice were detected by the presence of vaginal plugs. The day of vaginal plug detection was considered as half of the first pregnancy day.

**Generation of mouse embryos at the postimplantation stages of development**

Postimplantation embryos were recovered according to a standard protocol described by Monk [11]. Females were sacrificed by cervical dislocation. The abdominal cavity was opened, and the uterus with deciduomas was removed and placed in a Petri dish with cold phosphate buffered saline (PBS, Sigma-Aldrich). Next, the uterine horns were incised along the antimesometrial edge, exposing the decidual capsules with embryos and separating them from the mesometrial wall. The deciduomas were transferred into a clean Petri dish containing cold PBS. The decidual capsule was incised, capturing its mesometrial end, and the embryo was gently pushed into the solution. After that, the embryo was released from the amniotic sac by preparation needles and transferred to a clean Petri dish with PBS for washing. Embryos were fixed in 4% formaldehyde (PRS Panreac) in PBS at +4 °C overnight.

**Immunofluorescent staining of mouse embryos with antibodies to T-cadherin**

After fixation, embryos were washed 5 times for 20 min each in PBS containing a 0.2% Triton X-100 detergent (Sigma-Aldrich). To prevent nonspecific staining, the samples were placed into 1 : 10 normal goat non-immune serum (Sigma-Aldrich) and incubated at +4 °C on a shaker overnight. Then, the embryos were incubated in a 1 : 25 solution of the rabbit monoclonal antibody to mouse T-cadherin (BioDesign). As a control, non-immune rabbit IgG immunoglobulins (Abd Serotec) were used in a concentration equivalent to the concentration of specific antibodies. Incubation was carried out at room temperature under constant shaking for a day. Antibodies were washed in a 0.2% solution of Triton X-100 in PBS (three times for 20 min each at room temperature) and following that (in the fourth change of solution) on a shaker at +4 °C overnight. After washing, the embryos were placed in a solution of goat secondary antibodies conjugated with a fluorochrome Alexa FluorR 594 where they were kept on a shaker at room temperature for a day. Cell nuclei were additionally stained with a fluorescent dye DAPI (Sigma-Aldrich) in 1 : 1000 dilution for 30 min, then washed 3 times for 20 min each in 0.2% Triton X-100 in PBS, and left in the same solution at +4 °C on a shaker overnight. The next day, the embryos were mounted on a Aqua-Poly/Mount medium (Polysciences). The prepared samples were analyzed using a Leica SP5 confocal multiphoton microscope and Leica Application Suite Advanced Fluorescence 2.2.0 software (Leica Microsystems).

**Purification of plasmids for *in situ* hybridization**

Purification and isolation of plasmids were performed using a commercial EndoFreeR Plasmid Maxi Kit (Qiagen) according to the manufacturer’s protocol.

**Linearization of plasmids for** *in situ* **hybridization**

*In situ *hybridization with RNA probes to T-cadherin (sense and antisense) and Krox20 was performed on mouse embryos according to the previously developed technique [12].

To identify the expression of the T-cadherin mRNA in mouse embryos by *in situ *hybridization, the pFLCI plasmid (ImaGenes, Germany) with the inserted EST-sequence (expressed sequence tag) of the T-cadherin cDNA was used. The Bluescript KS plasmid with the inserted EST sequence of the Krox20 cDNA was used as a positive control. ESTs are short cDNAs used for detection of gene expression and are available in the GenBank database.

For the linearization, the plasmid DNA was treated with restriction enzymes: Not1 (Fermentas) for Krox20 and BamH1 (Fermentas) for T-cadherin. The composition of a linearization reaction mixture was as follows: 10× buffer, deionized water (Sintol), the plasmid (4 μg), and a restriction enzyme (Not1 or BamH1). The mixture was incubated at + 37 °C for 12 h. After incubation, DNA was purified using a commercial GFX PCR DNA kit and a Gel Purification Kit (GE Healthcare) according to the manufacturer’s protocol. Linearized and non-linearized plasmids were analyzed by electrophoresis in 1.2% agarose gel.

**Synthesis of a RNA-containing probe for** *in situ* **hybridization**

The reaction mixture for the synthesis of a digoxigenin- labeled RNA-containing probe included: 5× transcription buffer, RNase free deionized water, a linearized plasmid, a mixture of nucleotides labeled with DIG (10 mM ATP, 10 mM CTP, 10 mM GTP, 6.5 mM UTP, 3.5 mM DIG-11-UTP, pH 7.5, Roche), a RNase inhibitor (Merck Biosciences), and RNA polymerase. The mixture was incubated at +37 °C for 2 h. T3 RNA polymerase (Fermentas) was used to synthesize a RNA-containing probe from a linearized plasmid containing the Krox20 sequence. T7 RNA polymerase (Promega) was used to synthesize the RNA probe for the T-cadherin sense sequence (negative control) from the linearized plasmid, and T3 RNA polymerase (Fermentas) was used to synthesize the antisense RNA probe for detection of the T-cadherin mRNA. The resulting RNA probes were purified on a commercial RNAspin Mini column (GE Healthcare) according to the manufacturer’s protocol.

**Hybridization of mouse embryos *in situ***

Hybridization of mouse embryos was performed with the RNA probe (antisense) synthesized on the T-cadherin gene template. The antisense RNA probe (reverse sequence) enables detection of expression at the transcriptional level. The sense probe (direct sequence) does not bind to the mRNA in the cell, since it is non-complementary to the mRNA and is used as a negative control. The RNA probe synthesized on the *Krox20 *gene template, whose expression level is high in the central nervous system cells of mouse embryos at these stages of development, was used as a positive control.

Hybridization was performed in E8.75, E9.5 and E10.5 mouse embryos.

Embryos were fixed in 4% formaldehyde (PRS Panreac) in PBS, which also contained 1% Tween-20 (Sigma-Aldrich), at +4 °C overnight, then washed (2 × 5 min) with cold PBS, and fixed sequentially in solutions of increasing concentrations of methanol (25, 50, 75, and twice with 100%). After that, the embryos were frozen and stored for subsequent studies at –20 °C. Immediately prior to hybridization, the embryos were rehydrated in solutions of decreasing concentrations of methanol (75, 50, and 25%), washed three times in PBS, and treated with proteinase K at room temperature (Qiagen, at a concentration of 10 μg/mL in PBS). Then, the embryos were washed in PBS, fixed in 4% formaldehyde (PRS Panreac) in PBS for 20 min, and washed twice for 5 min in PBS. Next, the embryos were gradually transferred into a hybridization buffer containing 50% formamide (Sigma-Aldrich), 5× SSC buffer (stock solution: 20× SSC buffer containing 3 M NaCl, 0.3 M sodium citrate, pH 7.0), 0.1% Triton X-100 (DiaEm), 50 μg/mL heparin (Sigma-Aldrich), 1 mg/mL RNA from type IV yeast (Sigma-Aldrich), 5 mM EDTA (Applichem), 2% blocking solution (Roche), and 0.1% CHAPS (3-[(3-cholamidopropyl)-dimethylammonio]- 1-propanesulfonate) (Sigma-Aldrich). The embryos were incubated at room temperature in a (1 : 1) mixture of a hybridization buffer and PBS and then in the hybridization buffer. The embryos were left in the hybridization buffer at +65 °C overnight. In the morning, the embryos were placed in a fresh hybridization buffer, added with a RNA probe (0.5 μg of the probe per 1 mL of buffer), and incubated at +65 °C for 24 h. After that, the embryos were washed in the hybridization buffer (3 times for 30 min at + 65 °C) and then in a (1 : 1) mixture of the hybridization buffer and a MABT buffer at +65 °C for 30 min. The MABT buffer composition: 100 mM maleic acid (Sigma-Aldrich), pH 7.5, 150 mM NaCl, and 0.1% Tween-20 (Sigma-Aldrich). Next, the embryos were washed 3 times with the MABT buffer for 10 min at room temperature, placed in a blocking solution (2% blocking solution (Roche) containing 20% sheep serum (Abd Serotec) and MABT) at room temperature for 2 h, and then incubated in a 10% sheep serum solution containing 1 : 200 diluted anti-digoxigenin antibodies conjugated with alkaline phosphatase (Roche) at +4 °C for 12 h. After incubation with antibodies, the embryos were washed in MABT at room temperature and then in an NTMT buffer until staining. The composition of the NTMT buffer (per 1 mL): 100 mM Tris-HCl (Sigma-Aldrich), pH 9.5, 50 mM MgCl_2_ (Sigma-Aldrich), 100 mM NaCl, 0.1% Tween-20 (Sigma-Aldrich), 4.5 μL of NBT (4-nitro-blue tetrasodium chloride, Vector Laboratories), and 3.5 μL of BCIP (5-bromo-4-chloro-3-indolylphosphate, Vector Laboratories). The staining reaction was stopped by repeated washing in PBS. Then, the embryos were fixed in 4% formaldehyde (PRS Panreac) in PBS at room temperature for 2 h. Images of the embryos were produced using a stereomicroscope (Olympus SZX 16, AxioCam HRc camera, Carl Zeiss) and the Axio Vision 3.1 software.

## RESULTS

We evaluated the expression of T-cadherin at the E8.75–E11.5 stages of early embryonic mouse development using *in situ *hybridization and immunofluorescent staining of whole embryos in combination with confocal microscopy.

**Expression of T-cadherin in the embryonic mouse brain**

**Fig. 1:**
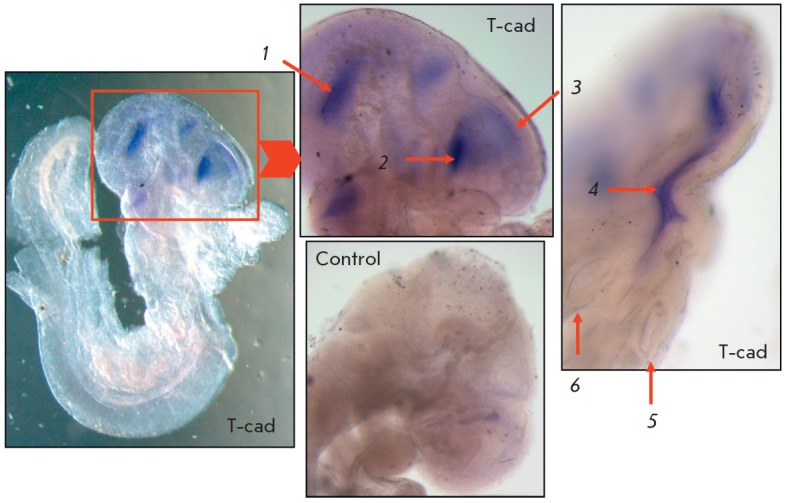
*In situ *hybridization of mouse embryos at the of E8.75 stage. Expression of T-cadherin mRNA (T-cad): *1 *– in the mesencephalon region;* 2 *– in the base of the developing optic vesicle;* 3 *– in the inner lining of the telencephalon; *4 *– in the myelencephalon; *5*,* 6 *– in the optic vesicles. No specific staining in the negative control (control). Magnification of 3.2, 5, and 6×

Expression of T-cadherin mRNA was detected in the developing brain, starting with the E8.75 stage, in particular, in the diencephalon and prosencephalon – in the inner lining of the telencephalon cavity (*Fig. 1*). The T-cadherin mRNA was also detected in the region of optic vesicles, at the transition of the diencephalon to the developing thalamencephalon. In the negative control (sense probe), nonspecific diffuse background staining in the prosencephalon was observed that was different from specific staining using the positive control and the antisense probe for T-cadherin.

**Fig. 2:**
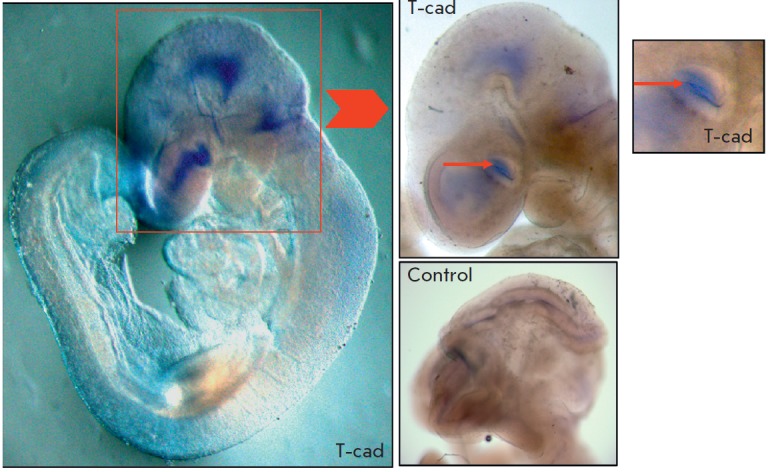
*In situ *hybridization of mouse embryos at the E9.5 stage. Staining of brain regions corresponds to the localization of T-cadherin mRNA (T-cad). Expression is observed in the base of the developing optic vesicles, in the parietal and occipital bend regions. Small arrows indicate the base of the developing optic vesicle. Control – the negative control. Magnification of 3.2, 5, and 6×

At the E9.5 stage, expression of the T-cadherin mRNA was detected in the prosencephalon, thickening olfactory placode, base of the optic vesicles, parietal bend region, and at the transition of the myelencephalon to the spinal cord (in the occipital bend region) (*Fig. 2*).

**Fig. 3:**
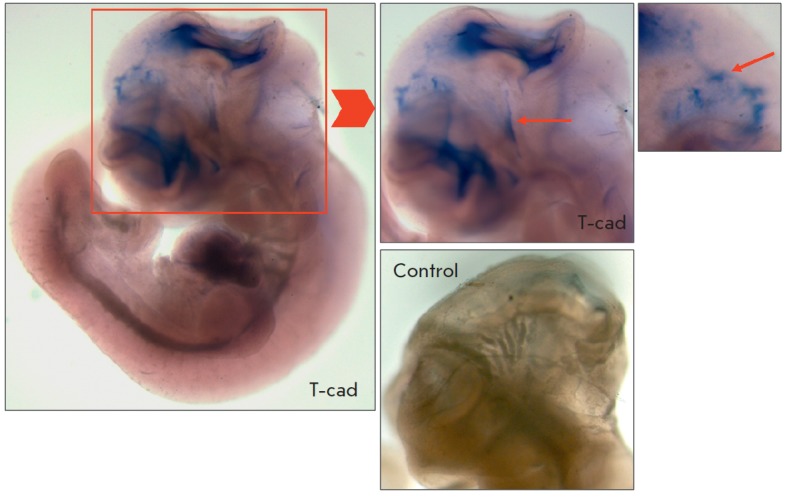
*In situ *hybridization of mouse embryos at the E10.5 stage. Intense expression of T-cadherin (T-cad) in the developing tectum and the lateral regions of the diencephalon. The arrow indicates specific staining of the choroid plexus in the telencephalon region. No specific staining in the negative control (control). Magnification of 3.2, 5, and 6×

At the E10.5 stage, expression of T-cadherin mRNA was observed in the mesencephalon and developing ependymal roof of the diencephalon and its lateral parts (*Figs. 3 *and *4*). Specific staining was also found in the choroid plexus of the telencephalon (*Fig. 3*).

No specific staining was found in the negative control. The specific staining pattern typical of the *Krox20* gene was observed in the positive control (*Figs. 3 *and *4*).

**Fig. 4:**
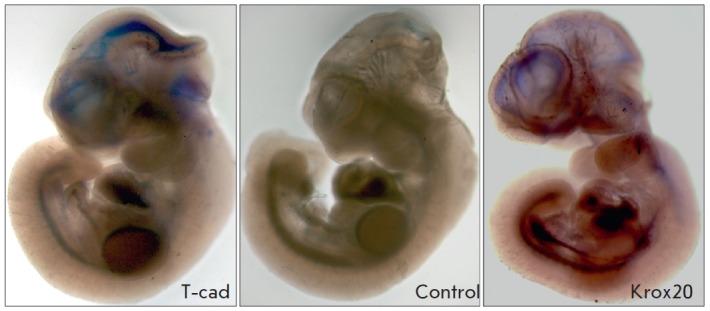
*In situ *hybridization of mouse embryos at the E10.5 stage. T-cad – specific staining of T-cadherin in the tectum (in the occipital bend region) and inner lining of the telencephalon; control – no specific staining in the negative control; Krox20 – staining of central nervous system structures in the positive control. Magnification of 3.2×

**Fig. 5:**
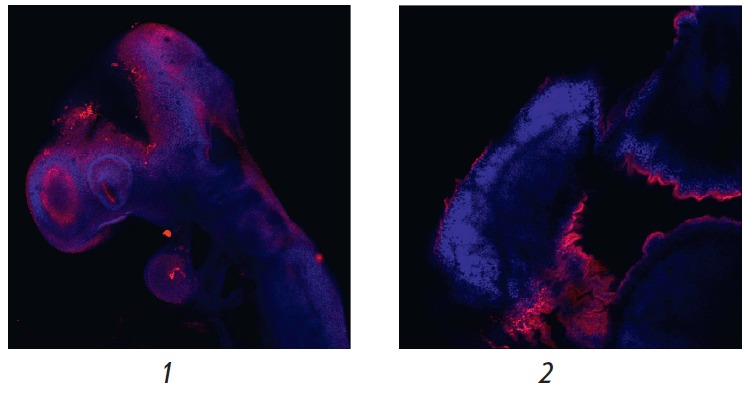
Immunofluorescent staining of mouse embryos at the E9.5 (-1-) and E12.5 (-2-) stages. Specific staining (red fluorescence) corresponds to T-cadherin expression in the linings of the brain at both stages; expression of T-cadherin in the developing optic vesicle in the E9.5 embryo. Blue fluorescence corresponds to nuclei additionally stained with DAPI. Magnification of 5×

The T-cadherin protein was detected by immunofluorescent staining with anti-T-cadherin antibodies in whole mouse embryos in combination with confocal microscopy. T-cadherin was detected starting with the E9.5 stage, with specific staining being observed in the linings of the developing brain (*Fig. 5*), including the base of the developing optic vesicles.

**Fig. 6:**
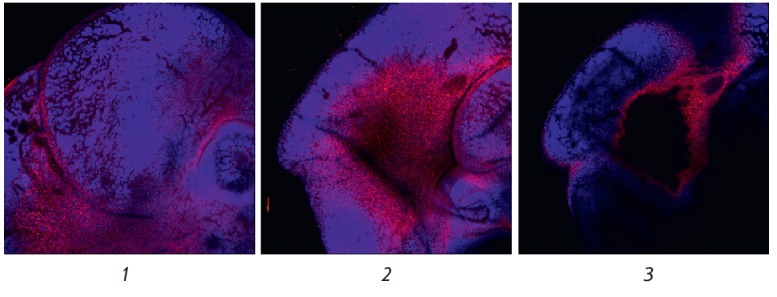
Immunofluorescent staining of mouse embryos at the E11.5 stage. Specific staining (red fluorescence) corresponds to expression of the T-cadherin protein. Blue fluorescence corresponds to nuclei additionally stained with DAPI.* 1 *– specific staining in the diencephalon region, as well as in the region of the developing eyecup; *2 *– specific staining in the mesencephalon and metencephalon region; *3 *– the same region as in *2 *– at another optical plane level. Magnification of 5×

The T-cadherin expression level in the inner lining of the brain was high, starting with the E11.5 stage: intense specific staining was observed in the diencephalon region, developing eyecup, as well as in the mesencephalon and metencephalon region (*Fig. 6*).

Therefore, these data indicate that T-cadherin expression at the mRNA level begins with the E8.75 stage and is detected in different parts of the embryonic brain. The T-cadherin protein is detected in embryos, starting with the E9.5 stage. The maximum intensity of T-cadherin expression was detected in the inner lining of the brain.

**Expression of T-cadherin in the embryonic heart**

**Fig. 7:**
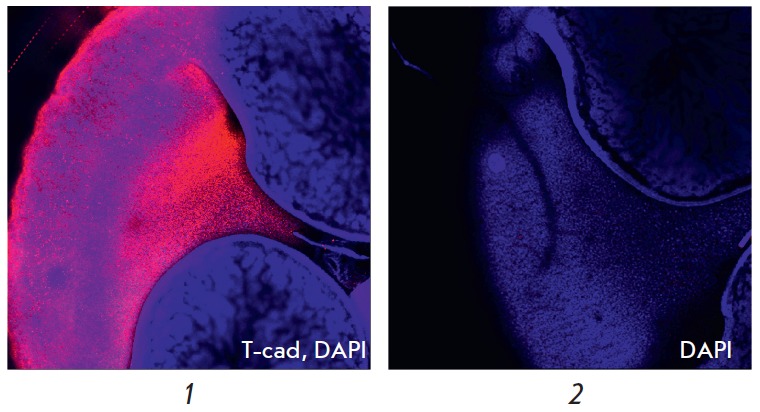
Immunofluorescent staining of mouse embryos at the E11.5 stage. Specific staining (red fluorescence) corresponds to expression of the T-cadherin protein (T-cad). Blue fluorescence corresponds to nuclei additionally stained with DAPI. *1 *– specific staining reflecting T-cadherin expression in the heart region; *2 *– control staining with antibodies to immunoglobulin G. Magnification of 5×

**Fig. 8:**
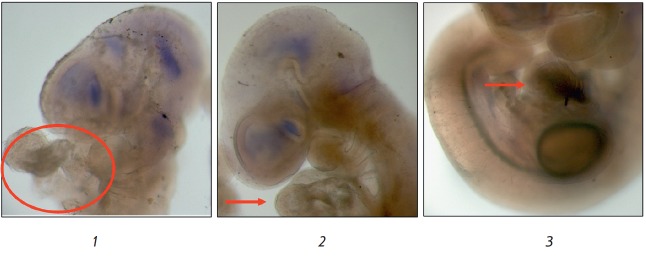
Lack of T-cadherin mRNA in the developing heart of mouse embryos at the E8.75–E10.5 stages (*1*, *2*, *3*). Arrows and the selected area indicate the developing heart region. Magnification of 5× (*1*,* 2*) and 6× (*3*)

In the mouse embryo heart, T-cadherin is expressed starting with the E11.5 stage (*Fig. 7*). No expression of either T-cadherin mRNA or T-cadherin in the developing heart was detected at the E8.75, E9.5 and E10.5 stages (*Fig. 8*).

## DISCUSSION

The obtained data indicate that T-cadherin mRNA in the developing brain is expressed starting with the E8.75 stage – in the inner lining of the telencephalon cavity and diencephalon. No expression of T-cadherin was detected until this stage. Active formation and growth of blood vessels are known to occur in the brain bend regions at the early developmental stages [13]. Probably, expression of T-cadherin at the E9.5 stage in these regions of the developing brain is associated with intensive angiogenesis and potential involvement of this protein in the regulation of directional growth of blood vessels in the same manner as it occurs during the growth of motoneuronal axons to their targets in the nervous system.

Later, at the E9.5 stage, the T-cadherin mRNA was identified in the prosencephalon, olfactory placode, base of the optic vesicles, and region of the parietal and occipital bends. The active formation and growth of blood vessels are known to occur in the brain bend regions at this stage, which suggests possible involvement of T-cadherin in vascularization of these structures [13]. Noteworthy, T-cadherin expression at the mRNA level in the optic vesicle region was detected at the E8.75 stage. We suppose that expression of T-cadherin at the base of the developing optic vesicles is associated with the epithelialization of the structures of the future eyecups; otherwise, T-cadherin could be involved in the choroid formation. However, further research is necessary to exactly determine the role of T-cadherin in the formation of these structures.

Later, at the E10.5 stage, intense staining corresponding to T-cadherin mRNA was detected in the mesencephalon, developing ependymal roof of the diencephalon, and its lateral parts. Specific staining was also found in the region of the choroid plexus of the telencephalon. The stained areas morphologically corresponded to the areas of the choroid plexus formation in the walls of the developing brain ventricular system.

The *in situ *hybridization results of T-cadherin expression detection at the protein level were confirmed by immunofluorescent staining of whole mouse embryos. Confocal microscopy combined with an image analysis enabled us to detect the T-cadherin protein in the linings of the developing brain, starting with the E9.5 stage. Expression of T-cadherin was also identified at the base of the developing optic vesicles, which corresponds to the *in situ *hybridization data. T-cadherin expression in the developing eyecups indicates the possible involvement of this protein in the choroid development.

Antibody staining of embryos revealed intense expression of T-cadherin in the inner lining of the brain, starting with the E11.5 stage. In particular, intense specific staining was observed in the diencephalon region, developing optic eyecup, as well as in the mesencephalon and metencephalon region. We suppose that T-cadherin is involved in the formation of the brain ventricular system, more specifically the choroid plexus in the ventricular walls, since the active formation of brain vessels is known to occur at this stage of embryonic development [13].

Therefore, the use of *in situ *hybridization and immunofluorescent staining in combination with confocal microscopy enabled us for the first time to detect T-cadherin in mouse embryos and identify the stage at which T-cadherin expression at the mRNA and protein level starts, as well as the morphological regions where the protein is expressed. In different parts of the developing brain T-cadherin expression at the mRNA level was detected starting from the E8.75 stage. Expression of the T-cadherin protein was detected starting from the E9.5 stage. The highest T-cadherin expression was observed in the inner lining of the brain, which suggests a possible involvement of T-cadherin in the formation of the choroid plexus in the ventricular walls of the developing brain.

*In situ *hybridization and immunofluorescent staining of whole mouse embryos revealed T-cadherin expression at the protein level in the heart, starting with the E11.5 stage. No expression of either T-cadherin mRNA or T-cadherin protein in the developing heart was observed at the E8.75, E9.5 and E10.5 stages.

Expression of T-cadherin in the embryonic heart, which was first identified at the E11.5 stage, reflects apparently the active formation and growth of the heart and its parts, as well as its vascularization [14].

Presumably, T-cadherin mRNA synthesis is activated between the E10.5–E11.5 stages of mouse embryo development; then, rapid and intense accumulation of the T-cadherin protein occurs. It is likely that T-cadherin is also involved in the formation of synaptic contacts in the developing cardiac conduction system.

Earlier, T-cadherin deficient mice were generated in the Ranscht laboratory [15]. These mice were viable and fertile, which typical for a wide range of knockout animals and indicates possible compensatory mechanisms implemented in embryogenesis. However, based on experiments in various animal models reproducing human cardiovascular diseases, T-cadherin was found to play an important role in the restoration of blood supply upon injury. By using an ischemia-reperfusion model, T-cadherin was demonstrated to perform the cardioprotective function, since the infarction size in the control mice was significantly less than that in T-cadherin-deficient animals [16, 17]. Based on a hindlimb ischemia model in these mice, T-cadherin was found to be necessary for a complete revascularization of ischemic muscles [18]. The present study suggests the role of T-cadherin as a guidance molecule regulating vascular growth during embryogenesis and is consistent with the results obtained in experimental animal models.

## CONCLUSIONS

The data on T-cadherin in the developing mouse brain and heart indicate that the onset of T-cadherin expression coincides with active formation and growth of blood vessels due to vasculogenesis and angiogenesis in the cardiovascular system and the brain [19]. These results suggest a role for T-cadherin as a molecule regulating the formation and directional growth of blood vessels during embryogenesis. As it was previously demonstrated, the mechanism by which T-cadherin regulates neuronal growth in the developing nervous system is based on homophilic recognition between T-cadherins on the contacting cells and their subsequent “repulsion” [2, 3]. Earlier, using *in vivo *and *in vitro *angiogenic models we had found that the same mechanism of homophilic interaction is used for the regulation of blood vessels growth [20]. We suggest that a similar mechanism involving T-cadherin could be utilized for the regulation of blood vessels growth in embryogenesis.

## Abbreviations

T-cad: T-cadherin
PBS: phosphate-buffered saline
